# Gait characteristics in community-dwelling older persons with low skeletal muscle mass and low physical performance

**DOI:** 10.1007/s40520-021-02061-0

**Published:** 2022-02-08

**Authors:** Yari Longobucco, Sebastian Krumpoch, Fulvio Lauretani, Valentina Angileri, Cornel Sieber, Emanuele Marzetti, Riccardo Calvani, Antonio Cherubini, Francesco Landi, Roberto Bernabei, Ellen Freiberger, Marcello Maggio

**Affiliations:** 1 Department of Medicine and Surgery, University of Parma, Via Antonio Gramsci 14, 43126 Parma, PR Italy; 2 Institute for Biomedicine of Aging, Friedrich-Alexander-University, Nuremberg, Germany; 3 Geriatric Clinic Unit, University Hospital of Parma, Parma, Italy; 4 Department of Rehabilitative Medicine, University Hospital of Parma, Parma, Italy; 5 Department of Geriatrics, Neuroscience and Orthopedics, Teaching Hospital “Agostino Gemelli”, Catholic University of the Sacred Heart School of Medicine, Rome, Italy; 6 Geriatrics and Geriatric Emergency Care, Italian National Research Center on Aging (IRCCS-INRCA), Ancona, Italy; 7 Department of Medicine, Kantonsspital Winterthur, Winterthur, Switzerland

**Keywords:** Gait, Gait analysis, Gait reserve capacities, Low muscle mass, Low physical performance, Older persons

## Abstract

**Background:**

Demographic changes in the western world entail new clinical approaches and challenges in older persons. Low skeletal muscle mass and low physical performance in older persons are both predisposing conditions for disability and obtaining knowledge in this cohort is essential.

**Aim:**

The primary aim of the study was to analyze a broader spectrum of gait characteristics within this specific population and differentiate them across different test conditions.

**Methods:**

Two centers participating at the SPRINTT project with hi-tech gait analysis available conducted a cross-sectional descriptive study on *N* = 115 community-dwelling older persons with low muscle mass and physical performance.

Reference values of 13 gait parameters were collected across different conditions: usual gait speed, fast gait speed, and usual gait speed while simultaneously naming animals.

**Results and discussion:**

This study shows the first spatio-temporal reference values in a community-dwelling older population composed of individuals with low skeletal muscle mass and low physical performance. In comparison to the normative spatio-temporal gait parameters in older persons reported in the literature, this population showed some differences. The mean gait speed was lower than 1 m/s, considered as a cutoff for vulnerable community-dwelling individuals, which corresponds to a greater risk of falls, hospitalization, and mortality. The stride length variability was higher, exposing to a greater risk of falling, and was also associated with a higher risk of developing cognitive decline.

**Conclusion:**

This study represents the first step in the development of quantitative reference values in community-dwelling older persons with low physical performance and low skeletal muscle mass.

## Introduction

Demographic changes in the western world entail new clinical approaches and challenges in older persons. Population over-60 is expected to rise from the current 900 million to 2 billion by 2050 [1], and since aging cannot be considered a homogeneous process, it is not always possible to meet a successful aging trajectory [2].

For this reason, it is important to introduce markers for identifying persons at risk of adverse events. For instance, gait speed is an indicator for overall health [3] and gait speed slower than 1.0 m/s [4] has been shown as predictive of many negative outcomes including frailty, mortality [5], mobility disability, hospitalization [6], falls and decreased quality of life [7]. This parameter has been used as a marker of physical performance. The term frailty, conceptualized as increased vulnerability and poor resolution of homeostasis facing a stressor event [8], has been operationalized by Fried et al. [9] with the presence of three or more of the following criteria: unintentional weight loss, self-reported exhaustion, weakness (low grip strength), low physical activity and, finally, slow gait speed.

The older persons’ ability to counter external stressors is represented by the intrinsic capacity, defined as “the composite of all the physical and mental capacities that an individual can draw on at any point in time” [10]. This concept overlaps with the reserve capacity, intended as the difference between fast and self-selected gait speed [11].

Gait speed also represents a marker of sarcopenia. In the revised recommendation by the European Working Group on Sarcopenia in Older Persons (EWGSOP2), a cutoff score of even 0.8 m/sec is defined as an indicator of severe sarcopenia [12].

In addition to the original definition of “loss of muscle mass” [13], the term sarcopenia includes other aspects, such as muscle strength and physical performance [12]. Sarcopenia has been acknowledged as an independent condition by the ICD-10-CM [14, 15] with an overall prevalence ranging between 6 and 22% in adults over-65 years and increasing with age [15].

Low skeletal muscle mass and low physical performance in older persons are both predisposing conditions for disability. These conditions are coming into the focus with numerous ongoing clinical trials, such as the SPRINTT study [16, 17]. This study has the double goal of finding a consensus on the identification of older persons with low skeletal muscle mass and low physical performance, and to test the effectiveness of a multifactorial intervention in this specific population living in the community.

As the percentage of this population is predicted to rise, obtaining additional knowledge in this cohort is essential. For all these reasons, the analysis of gait speed as cross-road marker of these conditions becomes of importance.

Gait speed covers only one aspect of gait performance, which can be influenced by numerous factors, such as age, trauma or illness [11]. The evaluation of specific gait parameters in relation to certain clinical phenomena should deserve more attention [18].

Despite some interesting recent perspectives [19], an accurate methodology is needed to assess gait speed and other characteristics of walking in this age group, because quantitative gait analysis can help to identify underlying pathological processes at an early stage and provide markers for intervention effects’ rate [11, 20, 21].

Hi-tech gait analysis in the laboratory (e.g., electronic walkways) provides a broader range of gait parameters [11], and several guidelines and recommendations gave already sufficient information regarding standardized test protocols and normative age and sex specific gait values [11, 20].

Two centers (Parma and Nuremberg) participating at the SPRINTT project and with the same hi-tech gait analysis system available in their laboratory decided to collect and merge data of community-dwelling older people with low physical performance and muscle mass.

Thus, the aims of the study were:

1. to analyze a broader spectrum of gait characteristics within this specific population and differentiate them across different test conditions, as suggested by the recommendations of the 2nd European GAITRite Meeting in Marseille in 2004 [22]: usual gait speed, fast gait speed and dual task;
2. to verify the presence of reserve capacity in persons with low physical performance and muscle mass;
3. to evaluate the contribution of physical function, assessed with the Short Physical Performance Battery (SPPB), on gait parameters;
4. to assess the contribution of age, sex and previous falls on gait parameters.

## Materials and methods

### Sampling and ethical approval

This is a sub-study of the “Sarcopenia and Physical fRailty IN older people multi-componenT Treatment strategies” (SPRINTT) clinical trial.

The SPRINTT trial is a phase III, single-blind, multicenter RCT (ClinicalTrials.gov identifier: NCT02582138) designed to compare the efficacy of a Multi-component Intervention (MCI) program (composed by physical activity, nutritional counselling plus dietary intervention, and an information and communication technology (ICT) intervention), versus a Healthy Aging Lifestyle Education (HALE) program, for preventing mobility disability in initially non-disabled older persons [16]. This study has enrolled a total of 1566 participants in 16 sites over 11 European countries [17].

The inclusion criteria were:

- Men and women aged ≥ 70 years;
- Short Physical Performance Battery (SPPB) score between 3 (included) and 9 (included);
- Able to complete the 400-m walk test within 15 min without sitting down, help from another person, use of a walker, or stopping for more than 1 min at a time;
- Presence of low muscle mass according to results from a Dual Energy X-Ray Absorptiometry (DXA) scan. In agreement with the Foundation for the National Institutes of Health Sarcopenia Project (FNIH) report 32, low muscle mass will be defined as: Body mass index-adjusted appendicular lean mass (aLM; i.e., the sum of lean mass from both arms and legs): < 0.789 in men, and < 0.512 in women, OR ii. aLM < 19.75 kg in men and < 15.02 kg in women [23];
- Willingness to be randomised to either intervention group and to follow the study protocol.

The exclusion criteria were:

- Unable or unwilling to provide informed consent or accept randomisation to either study group;
- Plans to relocate out of the study area within the next 2 years or plans to be out of the study area for more than 6 consecutive weeks in the next year;
- Residence in long-term care;
- Household member enrolled in the study;
- Current diagnosis of schizophrenia, other psychotic or bipolar disorder. Depression, per se, was not considered an exclusion criterion, allowing to assess its impact on the adherence to the intervention;
- Consumption of more than 14 alcoholic drinks per week;
- Difficulty communicating with the study personnel due to speech, language, or (noncorrected) hearing problems;
- Mini Mental State Examination (MMSE) lower than 24/30;
- Severe osteoarthritis (e.g., awaiting joint replacement) that would interfere with the ability to participate fully in either study arm;
- Cancer requiring treatment in the past 3 years, except for non-melanoma skin cancers or cancers that have an excellent prognosis (e.g., early stage breast or prostate cancer);
- Lung disease requiring regular use of supplemental oxygen;
- Inflammatory conditions requiring regular use of oral or parenteral corticosteroid agents;
- Severe cardiovascular disease (including New York Heart Association [NYHA] class III or IV;
- Parkinson’s disease or other progressive neurological disorder.

The centers of Nuremberg (Germany) and Parma (Italy) has realized this ancillary cross-sectional study, with the approval of the Scientific Committee of the SPRINTT Consortium. A subsample of 115 physical frail and sarcopenic older persons, 37 from Nuremberg and 78 from Parma, completed a gait analysis assessment.

### Assessment

Since this is an ancillary study of the SPRINTT project, we based on the definitions used by it. In this project the condition of low skeletal muscle mass and low physical performance is defined by the co-occurrence of three defining elements: low muscle mass (assessed by DXA), Short Physical Performance Battery (SPPB) summary score between 3 (included) and 9 (included), and absence of mobility disability, intended as the ability to complete the 400-m walk test [24]. More specifically, the SPPB is considered as a proxy of physical frailty [25], and low muscle mass is defined by the FNIH Criteria [23].

According to the SPRINTT study protocol, demographics, body mass index (BMI), history of falls in the last 12 months and symptoms associated with depression were recorded together with the cognitive and functional status [16].

The cognitive status was assessed with the Mini-Mental State Examination (MMSE) and the physical performance with the SPPB. To be included in the SPRINTT Project participants had to meet a MMSE score ≥ 24 and a SPPB score between 3 and 9. The SPPB represents one of the main physical tests able to predict mobility disability, and it represents a proxy of frailty [26]. Briefly, it is composed by three tasks: the ability to maintain balance in tandem, semi-tandem and side-to-side positions, the walking speed at usual pace over 4 m and the ability to stand from a seated position for 5 times. The score ranges from 0 to 12, and a participant is considered frail with a score between 3 and 9 (included). The inability to maintain Tandem’s position defined balance impairment. The SPPB was not originally designed to assess physical frailty, but the literature shows it is a test that accurately predicts disability across diverse populations, assessing the physical performance [26].

The reserve capacity, intended as the difference between fast and self-selected gait speed, was calculated. As specified by the SPRINTT protocol, symptoms of depression were assessed by the Center for Epidemiological Studies-Depression (CES-D) scale [27]. Skeletal muscle mass was obtained by Dual-energy X-ray absorptiometry (DXA), following the FNIH criteria by Studenski et colleagues [23]: appendicular lean mass (ALMcrude) (Inclusion criteria [kg]: ♀ < 15.02; ♂ < 19.75) and appendicular lean mass adjusted for BMI (ALM/BMI) (Inclusion criteria: ♀ < 0.512; ♂ < 0.789) were collected.

Gait analysis was performed using two electronic walkways (GAITRite Platinum, CIR Systems, Franklin NJ, USA) with embedded pressure sensors (Nuremberg: Gold walkway, 972 cm long, active electronic surface area 792 × 610 cm, total 29,952 pressure sensors, scanning frequency 60 Hz; Parma: 700 cm long, active electronic surface area 610 × 61 cm, total 23,040 pressure sensors, scanning frequency 60 Hz). The reliability and validity of the GAITRite system has been shown previously [28]. Both centers followed a standardized protocol in accordance with the recommendations of the 2nd European GAITRite Meeting in Marseille in 2004 [22]. After a safety and comprehension check by the assessment team, each participant performed a total of seven walks under three different test conditions: three walks in usual gait speed (single task), three walks in fast gait speed (single task) and the last in usual gait speed while simultaneously naming animals (dual-task). Participants started each walk at a line on the ground 1 m before the GAITRIte System and walked to a cone 1 m after the GAITRite System. The instruction for a walk in usual gait speed was: “Please walk in your usual gait speed until you reach the indicated cone.” The instruction for a walk at fast pace was: “Please walk as quickly and safe as possible without running until you reach the indicated cone.” The instruction for the dual-task was: “Please walk in your usual gait speed until you reach the indicated cone. As you walk, please try to name as many animals as possible.” For safety reasons, the tester always walked diagonally behind the participant. Participants wore their usual walking shoes and were permitted to use a straight cane as walking aid.

### Statistics

Mean and standard deviation of gait speed (m/s), step length (cm) and variability (%), stride length (cm) and variability (%), step width (cm) and variability (%), stride time (sec) and variability (%), step time (sec) and variability (%), cadence (step/min) and walk-ratio (cm/step/min) were collected. Values of the usual gait speed and the fast gait speed were calculated by the mean of the three walks performed.

The reserve capacity was assessed with the *t* test of Student.

Participants were divided into four groups based on the SPPB score (3–5, 6, 7, 8–9), and a p-for-trend ANOVA was performed to investigate possible differences in gait parameters between each group of SPPB score.

The contribution of age, sex and previous falls was evaluated with multiple linear regressions models. Spatio-temporal gait parameters were the dependent variables, with age, sex and previous falls as independent variables. All models were adjusted for BMI, ALMcrude and test center, which are well-known confounders.

Data were analyzed using Stata, version 13.0.

## Results

One-hundred and fifteen persons successfully performed the complete protocol.

The sample characteristics are reported in Table 1. The mean age was 79.83 (SD = 5.39) years, with a mean education of 9.71 (SD = 4.08) years, and 60% of participants were female. The average BMI was 28.47 and although the participants were all sarcopenic, in proportion women showed better values of appendicular lean mass than men. The mean MMSE score was 27.90 (SD = 1.71), and the score was confirmed after adjusting for age and educational level (27.53, SD = 1.62). The CES-D mean score was 4.83 (SD = 3.80). Regarding functional status, 41.7% of the participants reported one or more falls in the past year, consistent with the balance impairment found in 90.4% of the participants; likewise, the gait speed was 0.81 (SD = 0.22) m/s. The chair rise time was 18.21 s (SD = 6.34). These values translated into a SPPB mean score of 6.89 (SD = 1.16).

**Table 1:** Sample characteristics (*n* = 115)

Demographics	
Age (years), mean ± SD	79.83 ± 5.39
Sex (female), *n* (%)	69 (60)
Education (years), mean ± SD	9.71 ± 4.08
Anthropometry	
BMI (kg/m^2^), mean ± SD	28.47 ± 4.84
ALMcrude (kg), mean ± SD	
Men	18.41 ± 4.16
Women	16.65 ± 3.67
ALM/BMI, mean ± SD	
Men	0.66 ± 0.13
Women	0.59 ± 0.11
Cognitive status	
MMSE score, mean ± SD	27.90 ± 1.71
Age and educational level adjusted MMSE score, mean ± SD	27.53 ± 1.62
Depression	
CES-D score, mean ± SD	4.83 ± 3.80
Functional status	
Faller (yes), *n* (%)	48 (41.7)
SPPB summary score, mean ± SD	6.89 ± 1.16
Balance impairment (yes), *n* (%)	104 (90.4)
4-m gait speed (m/s), mean ± SD	0.81 ± 0.22
Chair rise time (sec), mean ± SD	18.21 ± 6.34

Table 2 shows the quantitative reference values of spatio-temporal gait parameters in older persons with low muscle mass and low physical performance, depending on different test conditions.

**Table 2:** Spatio-temporal gait parameters under different test conditions (mean ± SD)

	Usual speed	Fast speed	Dual-task
Gait speed (m/s)	0.99 ± 0.22	1.28 ± 0.28	0.95 ± 0.25
Step length (cm)	54.79 ± 8.46	61.44 ± 9.97	55.52 ± 9.70
Step length variability (%)	5.09 ± 2.31	4.39 ± 1.65	4.99 ± 3.21
Stride length (cm)	109.49 ± 17.42	122.78 ± 19.41	111.25 ± 19.35
Stride length variability (%)	4.20 ± 1.84	3.76 ± 1.33	3.67 ± 2.76
Step width (cm)	10.12 ± 3.42	9.83 ± 3.24	10.23 ± 3.79
Step width variability (%)	33.09 ± 16.00	33.14 ± 14.97	30.92 ± 16.10
Stride time (sec)	1.14 ± 0.13	0.98 ± 0.11	1.17 ± 0.20
Stride time variability (%)	8.43 ± 6.11	7.44 ± 5.53	8.31 ± 6.55
Step time (sec)	0.57 ± 0.07	0.49 ± 0.06	0.58 ± 0.09
Step time variability (%)	5.99 ± 2.76	5.43 ± 2.57	6.19 ± 4.01
Cadence (step/min)	106.89 ± 12.11	124.37 ± 14.26	105.41 ± 15.78
Walk-ratio (cm/step/min)	0.52 ± 0.08	0.50 ± 0.09	0.53 ± 0.09

The difference between fast and self-selected gait speed, proxy of reserve capacity, was 0.29 m/s (SD = 0.03) and statistically significant (*p* < 0.001).

The p-for-trend ANOVA results are shown in Table 3. We decided to not report the dual task data and the variability parameters for layout reasons, given the lack of statistically significant results. Regarding test conditions in usual gait speed, as expected, gait speed, step length and cadence showed higher values in the SPPB class of 8–9. Conversely, stride and step time showed lower values in this class. In the fast test condition, stride time and step time showed lower values in the SPPB class of 8–9, while cadence presented higher values.

**Table 3:** Spatio-temporal gait parameters by SPPB classes

Spatio-temporal gait parameters		SPPB score, mean ± SD				*p*-for-trend ANOVA	
		3–5 (*n* = 10)	6 (*n* = 13)	7 (*n* = 74)	8–9 (n = 18)	*F* [3, 111]	*p* value
Gait speed (m/s)	u	0.80 ± 0.18	0.93 ± 0.21	1.00 ± 0.22	1.07 ± 0.18	3.93	**0.01**
	f	1.10 ± 0.34	1.20 ± 0.25	1.30 ± 0.27	1.35 ± 0.23	2.38	0.07
Step length (cm)	u	49.40 ± 8.61	53.86 ± 9.98	54.80 ± 8.34	58.43	2.62	** < 0.05**
	f	56.61 ± 12.67	61.70 ± 12.14	61.30 ± 9.52	64.51 ± 8.04	1.37	0.26
Stride length (cm)	u	98.98 ± 17.18	108.11 ± 19.93	109.87 ± 17.24	114.79 ± 14.98	1.85	0.14
	f	113.52 ± 25.47	120.30 ± 19.44	122.90 ± 19.07	129.20 ± 16.01	1.51	0.22
Step width (cm)	u	10.62 ± 2.31	10.24 ± 3.02	9.88 ± 3.26	9.81 ± 2.58	0.20	0.89
	f	10.75 ± 3.42	9.72 ± 2.96	9.49 ± 2.83	9.89 ± 2.61	0.56	0.64
Stride time (sec)	u	1.25 ± 0.12	1.17 ± 0.10	1.13 ± 0.13	1.11 ± 0.14	3.26	**0.02**
	f	1.06 ± 0.13	1.01 ± 0.10	0.97 ± 0.11	0.96 ± 0.11	2.65	** < 0.05**
Step time (sec)	u	0.63 ± 0.06	0.59 ± 0.05	0.56 ± 0.06	0.55 ± 0.07	3.56	**0.02**
	f	0.53 ± 0.06	0.51 ± 0.05	0.48 ± 0.05	0.48 ± 0.06	2.68	** < 0.05**
Cadence (step/min)	u	96.93 ± 8.36	103.26 ± 7.83	108.08 ± 12.23	110.21 ± 13.20	3.56	**0.02**
	f	114.54 ± 14.53	119.75 ± 11.72	126.10 ± 14.01	126.06 ± 14.96	2.59	** < 0.05**
Walk-ratio (cm/step/min)	u	0.51 ± 0.08	0.52 ± 0.09	0.51 ± 0.08	0.54 ± 0.08	0.61	0.61
	f	0.49 ± 0.09	0.52 ± 0.10	0.49 ± 0.08	0.52 ± 0.09	0.79	0.50

*u* usual speed, *f* fast speed

Dual task was and variability parameters were excluded from the table due to the absence of significant results

The influence of age, sex and number of falls on spatio-temporal gait data is reported in Table 4. With increasing age, a significant change in almost all gait parameters was discovered. Exceptions were step width variability (dual-task condition), stride time variability and step time variability (usual and fast test condition). Women showed significant higher values over all tests conditions for step length, stride length and cadence.

**Table 4:** Linear regression analysis showing the association of spatio-temporal gait parameters with age, sex and falls

Spatio-temporal gait parameters		Age*			Sex* (Women)			Number of falls**		
		*β*	[95% CI]	*p* value	*β*	[95% CI]	*p* value	*β*	[95% CI]	*p* value
Gait speed (m/s)	u	0.006	[0.003;0.010]	** < 0.001**	0.089	[− 0.001;0.180]	0.052	− 0.001	[− 0.043;0.140]	0.965
	f	0.008	[0.004;0.012]	** < 0.001**	0.170	[0.060;0.280]	**0.003**	0.001	[− 0.050;0.052]	0.972
	d	0.007	[0.003;0.011]	** < 0.001**	0.103	[0.024;0.204]	**0.045**	0.019	[− 0.027;0.066]	0.416
Step length (cm)	u	0.388	[0.263;0.513]	** < 0.001**	4.792	[1.066;8.519]	**0.012**	0.422	[− 1.177;2.022]	0.602
	f	0.463	[0.320;0.606]	** < 0.001**	6.722	[2.471;10.973]	**0.002**	− 0.012	[− 1.828;1.803]	0.989
	d	0.373	[0.229;0.518]	** < 0.001**	5.217	[1.075;9.358]	**0.014**	1.245	[− 0.590;3.079]	0.181
Step length variability (%)	u	0.070	[0.037;0.102]	** < 0.001**	0.628	[− 0.292;1.549]	0.179	0.241	[− 0.176;0.658]	0.255
	f	0.086	[0.063;0.108]	** < 0.001**	0.883	[0.170;1.595]	**0.016**	0.245	[− 0.039;0.530]	0.090
	d	0.095	[0.051;0.139]	** < 0.001**	1.074	[− 0.161;2.308]	0.088	0.103	[− 0.460;0.666]	0.717
Stride length (cm)	u	0.733	[0.472;0.994]	** < 0.001**	10.122	[2.549;17.695]	**0.009**	1.013	[− 2.313;4.338]	0.547
	f	0.871	[0.592;1.151]	** < 0.001**	12.378	[4.127;20.629]	**0.004**	0.187	[− 3.366;3.740]	0.917
	d	0.751	[0.462;1.040]	** < 0.001**	10.411	[2.123;18.698]	**0.014**	2.524	[− 1.141;6.190]	0.175
Stride length variability (%)	u	0.037	[0.011;0.062]	**0.005**	0.223	[− 0.467;0.913]	0.523	0.218	[− 0.104;0.540]	0.183
	f	0.060	[0.041;0.078]	** < 0.001**	0.564	[0.003;1.125]	**0.049**	0.135	[− 0.101;0.371]	0.258
	d	0.071	[0.033;0.109]	** < 0.001**	0.704	[− 0.349;1.756]	0.188	0.113	[− 0.374;0.600]	0.647
Step width (cm)	u	0.081	[0.039;0.123]	** < 0.001**	0.540	[− 0.644;1.724]	0.368	− 0.415	[− 0.949;0.119]	0.126
	f	0.069	[0.030;0.109]	**0.001**	0.471	[− 0.640;1.581]	0.403	− 0.200	[− 0.711;0.310]	0.438
	d	0.067	[0.018;0.116]	**0.008**	0.853	[− 0.485;2.190]	0.209	− 0.410	[− 1.036;0.216]	0.197
Step width variability (%)	u	0.201	[− 0.001;0.402]	**0.049**	6.493	[1.242;11.743]	**0.016**	1.942	[− 0.457;4.341]	0.112
	f	0.237	[0.046;0.428]	**0.015**	5.715	[0.632;10.797]	**0.028**	2.149	[− 0.313;4.613]	0.087
	d	0.185	[− 0.038;0.409]	0.104	2.161	[− 3.848;8.171]	0.477	− 0.346	[− 3.258;2.567]	0.814
Stride time (sec)	u	0.012	[0.010;0.014]	** < 0.001**	0.110	[0.036;0.184]	**0.004**	0.017	[− 0.007;0.042]	0.169
	f	0.010	[0.009;0.012]	** < 0.001**	0.063	[− 0.003;0.129]	0.061	0.003	[− 0.018;0.024]	0.759
	d	0.012	[0.009;0.014]	** < 0.001**	0.083	[− 0.008;0.175]	0.075	− 0.002	[− 0.037;0.034]	0.927
Stride time variability (%)	u	− 0.054	[− 0.122;0.013]	0.114	− 0.322	[− 2.129;1.485]	0.725	0.810	[− 0.048;1.668]	0.064
	f	− 0.003	[− 0.067;0.060]	0.916	0.229	[− 1.443;1.902]	0.786	0.189	[− 0.626;1.004]	0.646
	d	− 0.048	[− 0.134;0.038]	0.274	− 1.473	[− 3.739;0.793]	0.200	0.159	[− 0.943;1.261]	0.775
Step time (sec)	u	0.006	[0.005;0.007]	** < 0.001**	0.053	[0.015;0.090]	**0.006**	0.008	[− 0.004;0.020]	0.176
	f	0.005	[0.004;0.006]	** < 0.001**	0.030	[− 0.002;0.062]	0.074	0.002	[− 0.008;0.013]	0.677
	d	0.005	[0.004;0.007]	** < 0.001**	0.046	[0.004;0.089]	**0.032**	− 0.001	[− 0.017;0.015]	0.905
Step time variability (%)	u	0.029	[− 0.006;0.064]	0.108	0.268	[− 0.662;1.199]	0.569	0.658	[0.226;1.090]	**0.003**
	f	0.044	[0.012;0.076]	**0.007**	0.138	[− 0.739;1.015]	0.756	0.297	[− 0113;0.707]	0.154
	d	0.024	[− 0.032;0.080]	0.402	0.287	[− 1.203;1.777]	0.703	0.162	[− 0.563;0.886]	0.659
Cadence (step/min)	u	0.945	[0.743;1.148]	** < 0.001**	11.261	[4.451;18.070]	**0.001**	− 0.300	[− 2.870;2.268]	0.817
	f	1.064	[0.827;1.301]	** < 0.001**	16.091	[8.466;23.716]	** < 0.001**	0.789	[− 2.143;3.721]	0.595
	d	0.967	[0.725;1.208]	** < 0.001**	12.309	[4.645;19.973]	**0.002**	1.370	[− 1.689;4.429]	0.377
Walk-ratio (cm/step/min)	u	0.004	[0.003;0.005]	** < 0.001**	0.042	[0.008;0.076]	**0.015**	0.008	[− 0.005;0.022]	0.242
	f	0.004	[0.003;0.005]	** < 0.001**	0.039	[0.002;0.075]	**0.036**	− 0.001	[− 0.015;0.014]	0.951
	d	0.004	[0.003;0.006]	** < 0.001**	0.035	[− 0.006;0.076]	0.092	0.007	[− 0.010;0.025]	0.425

In bold the significant *p* values

*u* usual speed, *f* fast speed, *d* dual task

*Adjusted for BMI, ALMcrude and test center

**Adjusted for age, sex, BMI, ALMcrude and test center

Women also showed higher values in usual and fast gait speed of step width variability and walk-ratio; greater values were also found in gait speed in the fast gait speed and dual task walks.

In the same way, we found higher values in step length variability and stride length variability in fast gait speed walks, in step time in usual and dual task walks, and finally in stride time in the usual gait speed walks. Only step time variability showed a significant association with the number of falls, in the usual gait speed walks.

## Discussion

Despite the relatively small sample, this study shows the first indicative spatio-temporal reference values in a well-characterized community-dwelling older population with low muscle mass and low physical performance.

In comparison to the normative spatio-temporal gait parameters in older persons of the Beauchet’s Consortium [20], who provided a minimum data set of spatio-temporal parameters assessed on a sample of 954 participants from 14 different countries, obtained only with a self-paced gait speed, our specific population showed some differences. The mean gait speed was lower than 1 m/s, considered as a cutoff for vulnerable community-dwelling individuals [4]. Slow gait speed corresponds also to a greater risk of falls, hospitalization, and mortality [11]. Stride time was similar between the two populations. Stride length was slightly lower in our cohort, while the stride length variability was higher. This last parameter may expose to a greater risk of falling, and is also associated with a higher risk of developing cognitive decline [11].

The significant difference of 0.29 m/s between fast and usual gait speed represents, in our sample of persons with low muscle mass and physical performance, a potential indicator of the presence of reserve capacity. Since this concept overlaps with the intrinsic capacity [29], it can be hypothesized that interventions targeting this value in persons with low muscle mass and physical performance could maintain independence and contribute to reverse or at least slow down the process leading to disability [30].

Our data show that the performance in the SPPB test seems to have an influence in some spatial–temporal gait parameters, which belong to pace factors (gait speed and step length) and rhythm factors (cadence, step time, stride time) [18]. While the differences in gait speed are probably explained by the fact that it is part of SPPB test, the other parameters may be of considerable importance, since there is evidence of a relationship between them and an increased risk of developing cognitive impairment [31]. Given the important clinical implications, the existence of this relationship should be further addressed. We should also underline that because of its descriptive nature, SPPB could be assessed in the primary care setting, the place of care closest to the living environment of the older person. If the patient’s results indicate they are physically frail, according to SPPB score, a gait analysis providing valuable additional information could be performed as a “second level” evaluation.

Interestingly, we found no significant relationships between the dual-task walk parameters and the SPPB. This may be due to the choice to use the MMSE score as inclusion criteria, and to the adopted minimum cutoff value. In addition, there is evidence that MMSE is not sensitive enough to exclude cognitive impairment [32]. Nevertheless, it seems that physical function alone is not sufficient to explain these differences, suggesting the need of combining motoric and a cognitive evaluation in the same setting.

A clinical meaningful difference of 0.18 m/s in mean usual gait speed was detected between the SPPB gait and the usual gait test condition of the gait analysis. This could be due to the different test protocols of both instruments (i.e., distance and end protocol). Another reason could be the different test burden on the participants. In fact, SPPB was collected during standard visits of the SPRINTT project, which are long (about 2 h) and involve other tests. The gait analysis was carried out in a separate, shorter and less demanding session. Our results demonstrate the need for the standardization of test protocols, as the advised cutoff speed of 0.8 m/s, an indicator for severe sarcopenia [12], was reached in the SPPB (0.81 m/s), but not in the gait analysis (0.99 m/s).

Finally, to guide this assessment, our data shows the importance of age and sex contribution in this specific population, contrary to what is reported in the literature [33], while previous falls did not display a significant role. The scarce impact of falls may be explained by the retrospective way of collection of this information with subsequent exposure to recall bias. If the contribution of age has been well documented [20], a special mention should be given to sex. Women showed higher values in step length, step width and stride length variabilities, which can expose females at a higher risk of falling and cognitive decline than male participants [11, 18, 31]. This last aspect is corroborated by the modifications in rhythm given by the higher levels of step and stride time [11, 18].

### Limitations and strengths

This study presents some limitations. First of all, this is a sub-study of SPRINTT trial, which was not originally designed to investigate these concepts. We acknowledge that the sample size is lower than previous studies, but given the descriptive nature of the manuscript, it should not preclude the quality of the results.

The participants are all Caucasian, and this may affect the generalizability of our results to other ethnicities.

Finally, this study used “proxies” of sarcopenia and physical frailty, instead of more traditionally used inclusion criteria, such as the SPPB for the physical performance instead the Fried phenotype for physical frailty, and standardized criteria of low skeletal muscle mass (FNIH criteria) instead of the updated version of EWGSOP (EWGSOP2) for sarcopenia, which are primarily based on muscle strength and then on muscle mass. This limitation is due to the fact that the SPRINTT protocol predates the publication of the EWGSOP2 criteria.

However, despite these limitations, to the best of our knowledge this is the first study to describe gait characteristics in community-dwelling older persons with low physical performance and low skeletal muscle mass. This population showed MMSE levels and CES-D values indicative of the absence of cognitive impairment and depression, conditions that can influence spatio-temporal gait characteristics. The multicentric nature of the study, together with the use of a common methodology with a standardized protocol, should ensure the reproducibility of results for Western Europe.

## Conclusions

This study represents the first step in the development of quantitative reference values in community-dwelling older persons with low physical performance and low skeletal muscle mass.

This typology of the participants requires special attention, due to its specific characteristics and the high risk of adverse events. The functional status and sex are the most relevant aspects that should be taken into account during the assessment of the spatio-temporal gait parameters in this population.

It was demonstrated that gait variability could be treated by physical activity, in different frailty-related scenarios [34–36]. The knowledge of reference values may be helpful to develop specific exercise programs for this type of patients.

However, further studies will be necessary to reach a full consensus on normative gait parameters.
